# Collared cemented femoral stems are associated with lower periprosthetic femoral fracture rates: A systematic review and subgroup meta‐analysis

**DOI:** 10.1002/jeo2.70969

**Published:** 2026-10-28

**Authors:** Pierre Laboudie, Mattia Blancatto

**Affiliations:** ^1^ Clinique du Sport Bordeaux‐Mérignac, Groupe Vivalto Mérignac France; ^2^ Department of Orthopaedics and Traumatology University of Milan, ASST Lecco Milan Italy

**Keywords:** cemented stem, collared stem, collarless stem, periprosthetic femur fracture, polished stem

## Abstract

**Purpose:**

Periprosthetic femoral fracture (PFF) is a severe complication after hip arthroplasty. Cemented femoral stems have been used to reduce this risk, while collars have been associated with lower fracture rates in cementless stems. However, the role of a collar in cemented femoral stems remains unclear. This systematic review and meta‐analysis compared postoperative PFF rates between collared and collarless cemented femoral stems.

**Methods:**

Nine comparative cohorts directly comparing collared and collarless cemented stems were included in the odds ratio (OR) meta‐analysis. Eligible single‐arm cohorts were additionally included in pooled incidence analyses. Random‐effects models were used throughout. Predefined subgroup analyses were performed according to surgical indication and stem surface finish, including a polished‐stem subgroup comparing polished collared with polished collarless designs.

**Results:**

Collared stems were associated with a lower postoperative PFF rate (OR, 0.28; 95% confidence interval [CI], 0.17–0.49; *p* < 0.00001; *I* ^2^ = 71%). Pooled PFF incidence was 0.51% for collared stems and 2.27% for collarless stems. This association was observed in elective arthroplasty cohorts (OR, 0.30; 95% CI, 0.15–0.63) and femoral neck fracture cohorts (OR, 0.28; 95% CI, 0.20–0.38). In the polished‐stem subgroup, analysis showed a lower odds of PFF with polished collared stems (OR, 0.17; 95% CI, 0.01–2.86; *p* = 0.22). Pooled incidence was 0.65% for polished collared stems and 2.34% for polished collarless stems.

**Conclusions:**

Collared stems were associated with lower PFF rates. This association was observed in both elective arthroplasty and femoral neck fracture cohorts. Polished collared designs also showed lower PFF rates than polished collarless designs. Substantial heterogeneity and confounding by other stem characteristics limit interpretation.

**Level of Evidence:**

Level III.

## Graphical abstract

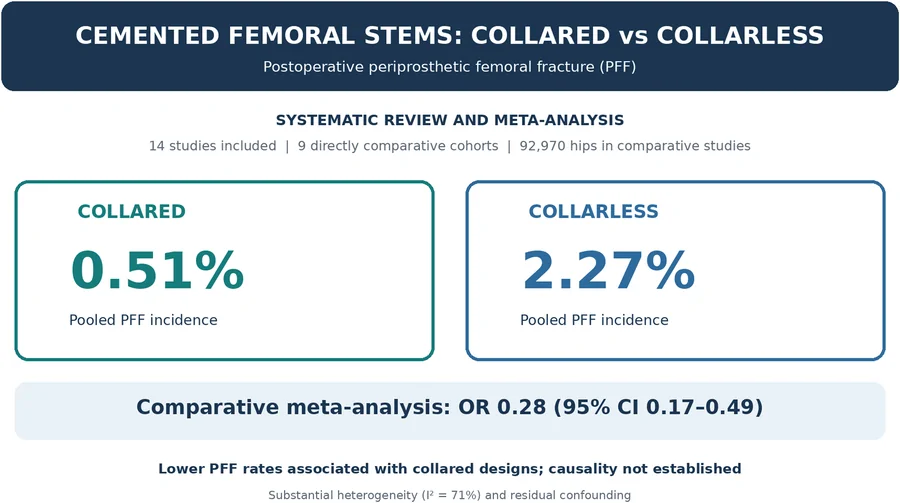

**Abbreviations**
CB: composite‐beam
CI: confidence interval
CK: Charnley–Kerboull
HA: hemiarthroplasty
NOS: Newcastle–Ottawa Scale
OR: odds ratio
PFF: periprosthetic femoral fracture
PRISMA: Preferred Reporting Items for Systematic Reviews and Meta‐Analyses
THA: total hip arthroplasty
TS: taper‐slip

## INTRODUCTION

Periprosthetic femoral fracture (PFF) is an increasingly important complication of total hip arthroplasty (THA) and hemiarthroplasty (HA), with its absolute incidence expected to rise by approximately 4%–5% per decade as the volume of hip arthroplasty continues to increase [1, 21, 33]. These fractures carry a severe clinical burden with a high rate of complications and a mortality rate around 15% within 1 year [21, 22]. The prevention of this complication therefore represents a major clinical and public health priority.

Risk factors are multifactorial, and many of these are non‐modifiable [3, 26]; however, implant‐related factors can be controlled by the surgeon's choice. Notably, the mode of femoral fixation appears to influence fracture risk: Large registry data indicate that using a cemented femoral stem is associated with a lower rate of early PFF even though cemented stems come with inherent risks such as bone cement implantation syndrome [8, 36, 39]. However, cemented stems encompass different design philosophies and techniques. For example, taper‐slip (TS) stems are collarless and polished, achieving fixation via controlled subsidence within the cement mantle in order to protect the cement bone interface, whereas composite‐beam (CB) stems have a rough surface finish to bond to the cement and incorporate a collar to engage the calcar [10, 40]. The French paradox cementing technique represents another fixation philosophy and uses a highly polished, collared, canal‐filling stem [17]. Evidence from cementless THA suggests that a collar can be protective against early PFF [19, 20, 30, 35, 43]. By analogy, it is hypothesized that even in cemented stems, a collar might confer a similar protective effect through related biomechanical mechanisms, even though collar status is closely associated with different stem geometries, surface finishes and fixation philosophies.

The primary aim of this systematic review and meta‐analysis was to compare postoperative PFF rates between collared and collarless cemented femoral stems. It was hypothesized that collared cemented femoral stem designs would be associated with lower postoperative PFF rates than collarless designs. To partly address differences in patient populations, elective primary arthroplasty and femoral neck fracture cohorts were analysed separately. Because non‐polished CB stems were consistently collared, this design category could not distinguish the association of collar status from that of other stem characteristics. An additional exploratory comparison was performed among polished cemented stems, for which both collared and collarless designs were available, while recognizing that geometry and fixation philosophy could still differ.

## MATERIALS AND METHODS

### Protocol and registration

This systematic review was conducted and reported in accordance with the Preferred Reporting Items for Systematic Reviews and Meta‐Analyses (PRISMA) 2020 guidelines [31].

### Literature search strategy

A systematic search was conducted for studies published from 1 January 2000 through 30 June 2026 in MEDLINE via PubMed, Embase via Elsevier, Web of Science Core Collection via Clarivate and the Cochrane Library. The search combined the terms ‘periprosthetic femoral fracture’, ‘hip arthroplasty’, ‘cemented stem’, ‘collared cemented stem’, ‘collarless cemented stem’, ‘composite‐beam stem’ and ‘taper‐slip stem’. Reference lists of eligible articles were also screened. Citation searching was performed for all full‐text manuscripts to identify manuscripts that were not found in initial searches. Abstracts and the full texts were screened by two authors (M.B. and P.L.) independently, and disagreements at each stage were settled by consensus. The risk of bias/quality of studies was assessed using the Newcastle–Ottawa scale (NOS) [32] (Appendix 1) by two authors (M.B. and P.L.). Assessments were performed independently, and disagreements were resolved by discussion until consensus was reached. Risk‐of‐bias findings were considered when interpreting the pooled estimates, particularly in relation to selection bias and residual confounding.

The following variables were collected for each study: publication year, study design, sample size, femoral stem model and manufacturer, patient characteristics, collar status, surface finish, stem design philosophy, type of arthroplasty, surgical indication (elective vs. femoral neck fracture), follow‐up duration, number of postoperative PFF and Vancouver classification distribution. PFF events were counted over the follow‐up period reported by each study. Pooled incidences were not standardized to a common follow‐up time or calculated using person‐years. When available, data were extracted separately for elective primary arthroplasty and femoral neck fracture cases and for polished versus non‐polished cemented stems. When PFF incidence was reported only as a percentage, the number of events was derived from the sample size.

### Inclusion and exclusion criteria

#### Inclusion

Peer‐reviewed clinical studies of comparative or observational design (randomized trials, non‐randomized comparative studies, prospective or retrospective cohorts, registry analyses) were eligible if they met all the following:

- Population: >18 years old.
- Surgery: Primary elective THA or THA/HA for femoral neck fracture.
- Implant: Cemented stem with clearly specified stem type, collar status and surface finish (polished vs. non‐polished).
- Outcomes: Report of PFF rate, type of PFF according to the Vancouver classification [24].
- Follow‐up: 1 year or more

#### Exclusion

Studies were excluded if they met any of the following:

- Revision arthroplasty as the index procedure.
- Intraoperative fractures: They were excluded to ensure the homogeneity of the results. Firstly, their mechanism is different, depending mainly on implantation manoeuvres and not reflecting the behaviour of the stem under load at follow‐up. Secondly, combining intra‐ and postoperative events would increase heterogeneity and bias, reducing the internal validity of the analysis of the effect of the collar on postoperative PFFs.
- Non‐original research (reviews, guidelines, technical notes, case reports).
- Abstracts or unpublished data without a peer‐reviewed full text.

### Analyses

#### Primary analyses

The primary comparative analysis was restricted to studies directly comparing collared and collarless cemented femoral stems. For these comparative studies, the association between collar status and postoperative PFF was assessed using odds ratios (ORs) with 95% confidence intervals (CIs). Both common‐effect Mantel–Haenszel and random‐effects inverse‐variance models were calculated, with the random‐effects model used as the primary model because clinical and methodological heterogeneity was expected across studies.

In addition to comparative OR analyses, pooled incidence analyses were performed separately for collared and collarless cemented stems. For these incidence analyses, both comparative study arms and eligible single‐arm cohorts were included, provided that the number of postoperative PFFs and the corresponding number of hips at risk were available. Incidence proportions were pooled with 95% CIs using a random‐effects model. Thus, comparative studies contributed to both the OR meta‐analysis and the pooled incidence analyses, whereas single‐arm studies contributed only to pooled incidence estimates and were not included in the comparative OR analysis.

### Subgroup and sensitivity analyses

Subgroup analyses were performed according to surgical indication (elective primary arthroplasty versus femoral neck fracture) and stem surface finish. Because non‐polished CB stems were consistently collared, the surface‐finish comparison was restricted to highly polished cemented stems. For a study to contribute to a comparative subgroup analysis, the number of postoperative PFF events and the number of hips at risk had to be available separately for collared and collarless stems within that subgroup. Studies reporting only combined data across indications or surface finishes were not assigned to a subgroup unless these counts could be calculated unambiguously from the published data. No events or denominators were apportioned using estimated proportions.

### Statistical analysis

Quantitative synthesis was conducted in R (Version 3.4.2) using the *meta* package. For comparisons between collared and collarless stems, ORs with 95% CIs were pooled using a random‐effects inverse‐variance model. A common‐effect Mantel–Haenszel model was used as a sensitivity analysis. Between‐study variance (*τ* ^2^) was estimated by restricted maximum likelihood for OR meta‐analyses. For the separate pooled incidence analyses, proportions were combined using a random‐effects model with the Freeman–Tukey double‐arcsine transformation; *τ* ^2^ was estimated by the DerSimonian–Laird method. Studies reporting zero PFF events were retained, with a continuity correction of 0.5 applied where required for variance calculation. Pooled estimates and 95% CIs were back‐transformed to proportions. Heterogeneity was assessed using Cochran's *Q* test (*p* < 0.10) and quantified by *I* ^2^. Potential publication bias was assessed by visual inspection of a funnel plot of the comparative studies. Differences in Vancouver fracture‐type distributions were tested using the *χ* ^2^ test. Statistical significance was set at *α* = 0.05.

## RESULTS

### Study characteristics

After duplicate removal and title/abstract screening, 37 full‐text articles were assessed for eligibility. Twenty‐three were excluded, leaving 14 studies for inclusion (Figure 1). These comprised nine comparative cohorts directly comparing collared versus collarless cemented femoral stems (Table 1) and five single‐arm studies evaluating a single stem design (Table 2). The nine comparative cohorts comprised 92,970 hips in total, including 60,755 (65.4%) hips in collared‐stem groups and 32,215 (34.6%) hips in collarless‐stem groups, with individual study sizes ranging from 131 to 82,837 hips. Follow‐up in comparative cohorts ranged from 20 months to 10 years. The five single‐arm studies included 3989 hips, with follow‐up durations ranging from 2.6 to 21.5 years.

**Figure 1:**
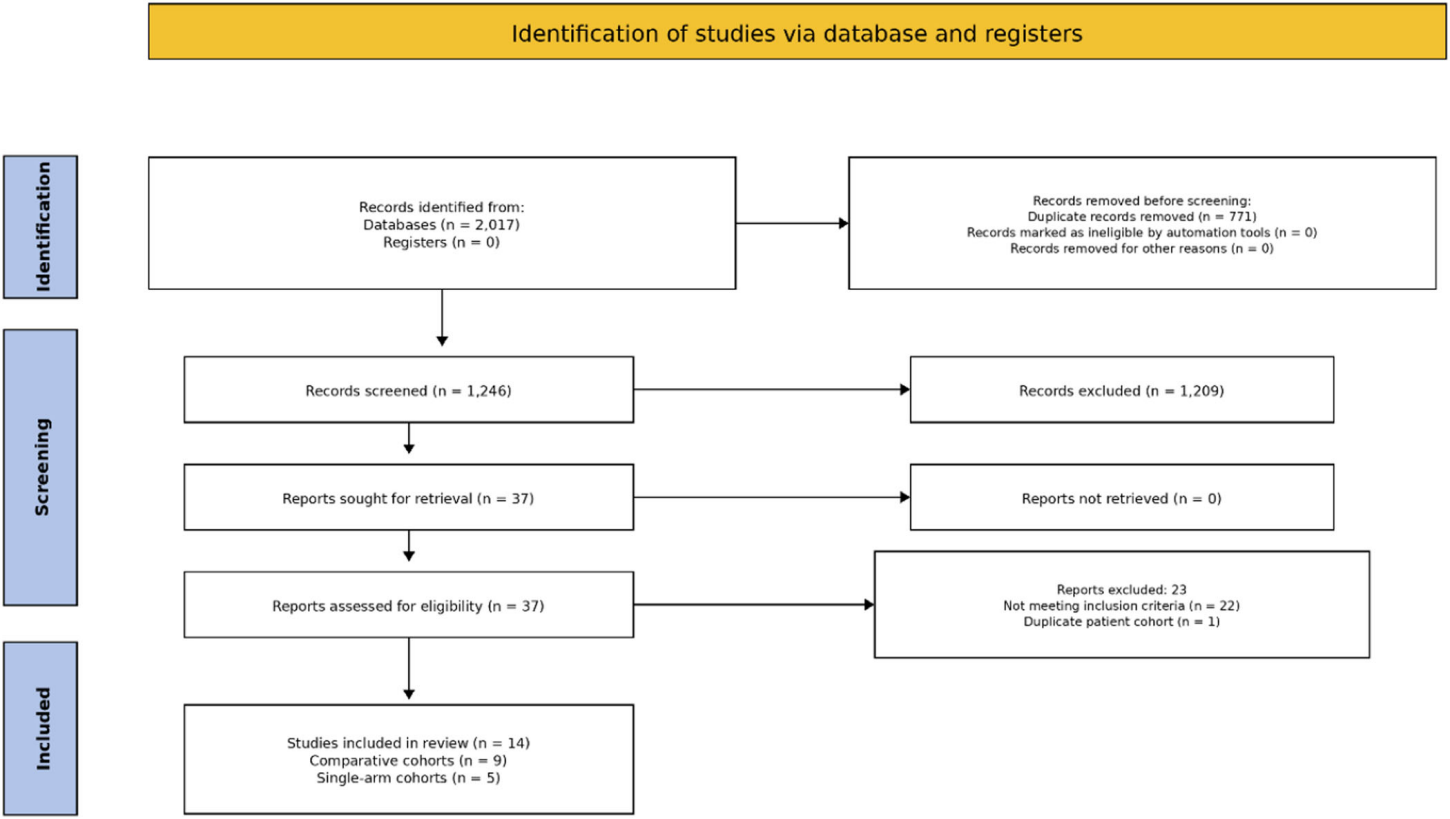
Flow diagram of study selection for the systematic review, showing the 37 full‐text articles assessed, 23 exclusions and 14 studies included (nine comparative and five single‐arm).

**Table 1:** Summary of comparative studies evaluating the incidence of periprosthetic femoral fractures (PFFs) in cemented collared versus collarless femoral stems, including registry, multicenter and single‐centre cohorts.

Author	Study type	*N*	Stems type (*N*)	PFF rate	Mean follow‐up
Chatziagorou et al. *Acta Orthop*. 2019 [6]	Registry analysis (national registry)	79,813	Collared non‐polished (Lubinus SPII): 52.625	167 → 0.3%	5.6 years
			Collarless: (Exeter) 27.188	298 → 1.1%	
Mellner et al. *Eur J Trauma Emerg Surg*. 2021 [25]	Retrospective multicenter cohort	2528	Collared non‐polished (Lubinus SPII): 1.202	9 → 0.7%	3.9 years
			Collarless (Exeter): 1.326	30 → 2.3%	
Nakagawa et al. *BMC Musculoskelet Disord*. 2025 [29]	Retrospective cohort (single‐centre)	131	Collared non polished(Charnley Elite Plus): 89	0 → 0%	14.3 years
			Collarless (C‐Stem): 42	3 → 7.1%	
Josefsson et al. *J Exp Orthop*. 2025 [14]	Prospective cohort (single‐centre)	1076	Collared non‐polished (Lubinus SPII): 534	5 → 0.9%	6 years
			Collarless (CPT): 505	23 → 4.6%	
Rames et al. *J Arthroplasty*. 2022 [34]	Retrospective cohort (single‐centre)	1306	Collared non‐polished (Omnifit EON): 798	34 → 4.3%	6.1 years
			Collarless (Exeter): 508	18 → 3.5%	
Miyamoto et al. *Injury*. 2022 [27]	Retrospective cohort (single‐centre)	1082	Collared polished: 203 (Charnley–Kerboull) Collared non‐polished (Charnley Elite Plus, KC, ISOFAR, PHS): 157	Polished: 0 → 0% Non‐polished: 1 → 0.6%	9.4 years
			Collarless (Trilliance, Exeter, C‐Stem, CPT, SC): 722	10 → 1.4%	
Scott et al. *J Arthroplasty*. 2018 [37]	Retrospective cohort (single‐centre)	1460	Collared non‐polished (VerSys): 1275	3 → 0.23%	1.8 years
			Collarless (CPT): 185	3 → 1.63%	
Mukka et al. *Acta Orthop*. 2016 [28]	Prospective cohort (multicenter)	979	Collared non‐polished (Lubinus SPII): 424	1 → 0.2%	1.7 years
			Collarless (CPT): 555	21 → 3.8%	
Chen et al. *J Arthroplasty*. 2025 [7]	Retrospective registry cohort	4631	Collared non polished(Summit, VerSys, Synergy, Endurance, Accolade C, Echo FX): 3.447	10 → 0.29%	6 years
			Collarless (C‐Stem, CPCS, CPT, Exeter): 1.184	14 → 1.18%	

**Table 2:** Summary of single‐arm cohort studies reporting periprosthetic femoral fracture (PFF) rates for cemented femoral stems, including exclusively collared and exclusively collarless taper‐slip designs.

Author	Year	Study type	*N*	Stem type	PFF rate	Follow‐up
Turnbull et al. [42] *Eur J Orthop Surg Traumatol*	2024	Single‐arm cohort (prospective)	1000	Collared non‐polished: Lubinus SP II	3 → 0.3%	21.5 years
Laboudie et al. [16] *Bone Jt J*	2024	Single‐arm cohort (retrospective)	416	Collared polished: Charnley–Kerboull (AmisK)	1 → 0.24%	2.6 years
Baryeh et al. [2] *Arch Orthop Trauma Surg*	2023	Single‐arm cohort (prospective)	500	Collarless: C‐Stem	7 → 1.4%	15 years
Brodén et al. [5] *Acta Orthop*	2015	Single‐arm cohort (prospective)	1403	Collarless: CPT	47 → 3.3%	4 years
Laboudie et al. [18] *Arthroplasty Today*	2026	Single‐arm cohort (retrospective)	670	Collared polished: Charnley–Kerboull (AmisK)	10 → 1.49%	2.6 years

Among the included studies, the following stem designs were evaluated:

Collared stems:

- Lubinus SP II/SP2 (Waldemar Link)—non‐polished
- Charnley Elite Plus (DePuy Synthes)—non‐polished
- Charnley–Kerboull (CK) (AmisK Medacta, Initiale Amplitude, Biomet)—polished
- VerSys Heritage (Zimmer Biomet)—non‐polished
- Omnifit EON (Stryker)—non‐polished
- Summit (DePuy Synthes)—non‐polished
- Synergy (Smith & Nephew)—non‐polished
- Endurance (DePuy Synthes)—non‐polished
- Accolade C (Stryker)—non‐polished
- Echo FX (Biomet)—non‐polished
- KC (Kyocera)—non‐polished
- ISOFAR (B. Braun–Aesculap)—non‐polished
- PHS (Kyocera)—non‐polished

Collarless stems:

- Exeter (Stryker)—polished
- CPT (Zimmer Biomet)—polished
- C‐Stem/C‐Stem AMT (DePuy Synthes)—polished
- Trilliance (B. Braun–Aesculap)—polished
- SC (Kyocera)—polished
- CPCS (Smith & Nephew)—polished

### Primary analysis

Nine comparative studies were included in the meta‐analysis. The pooled OR for postoperative PFF with collared versus collarless cemented stems was 0.28 (95% CI, 0.17–0.49; *p* < 0.00001), with substantial between‐study heterogeneity (*I* ^2 ^= 71%; *p* = 0.0006) (Figure 2).

**Figure 2:**
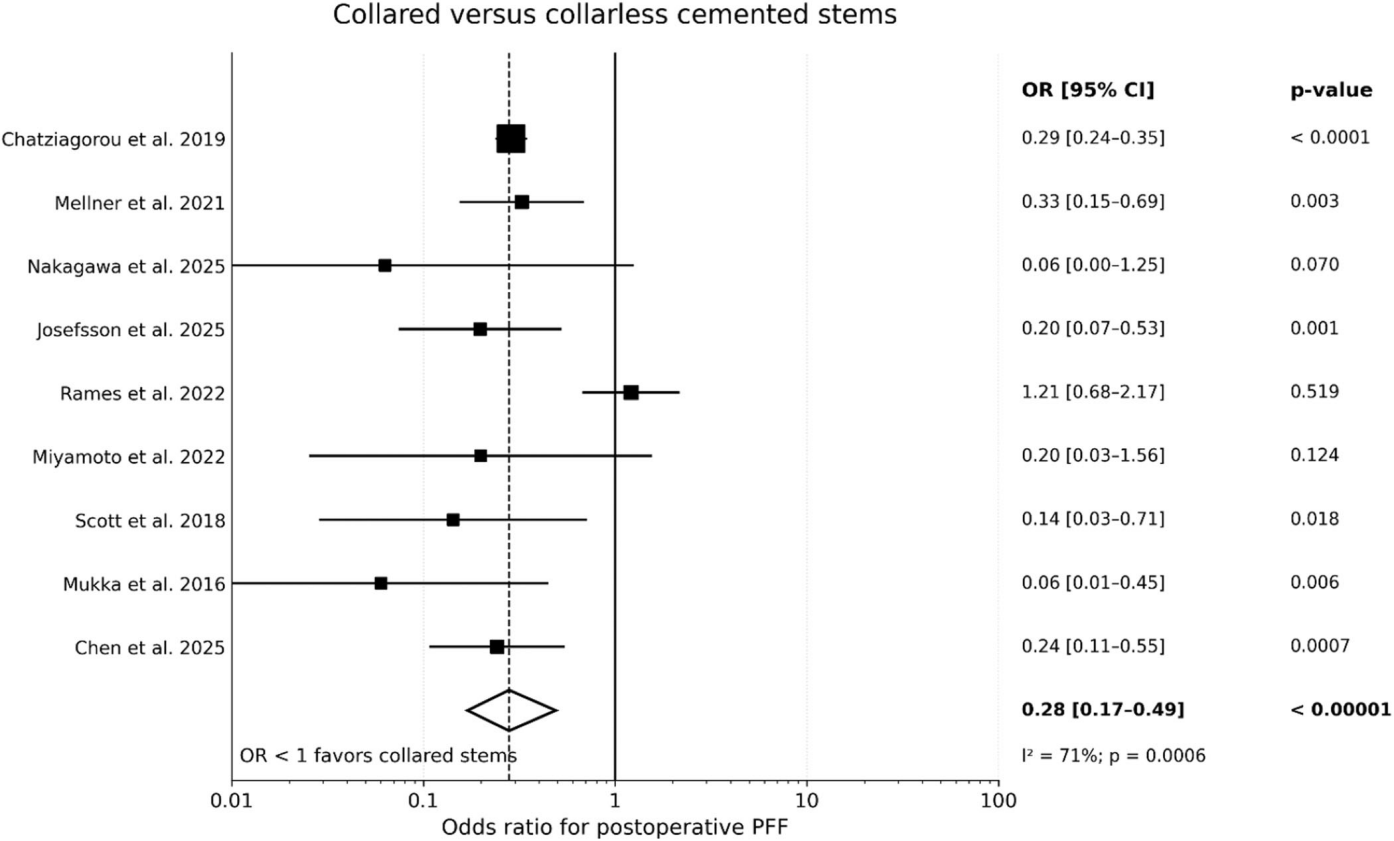
Forest plot showing the odds ratio (OR) and 95% CIs for postoperative PFF comparing collared versus collarless cemented stems. The pooled random‐effects OR was 0.28 (95% CI: 0.17–0.49), represented by the diamond. OR < 1 favours collared stems. CI, confidence interval; PFF, periprosthetic femoral fracture.

The pooled incidence of PFF was 0.51% (95% CI, 0.22%–0.90%; *I* ^2 ^= 87.8%) for collared stems and 2.27% (95% CI, 1.46%–3.23%; *I* ^2^ = 90.0%) for collarless stems (Figures 3 and 4).

**Figure 3:**
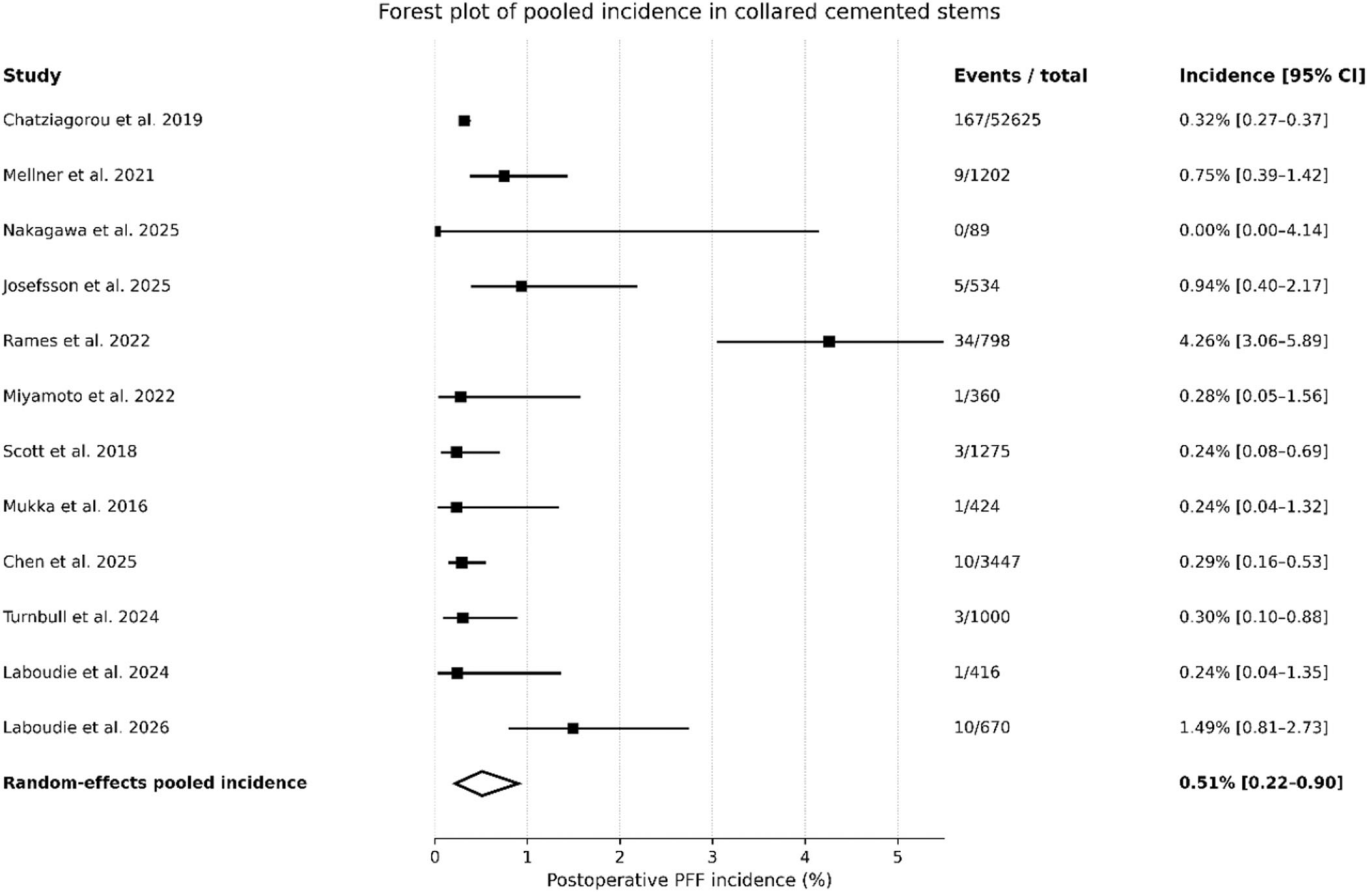
Forest plot of the pooled incidence of postoperative PFF in the collared cemented stem group. The combined incidence was 0.51%, represented by the diamond. Each line corresponds to an individual study and its CI. CI, confidence interval; PFF, periprosthetic femoral fracture.

**Figure 4:**
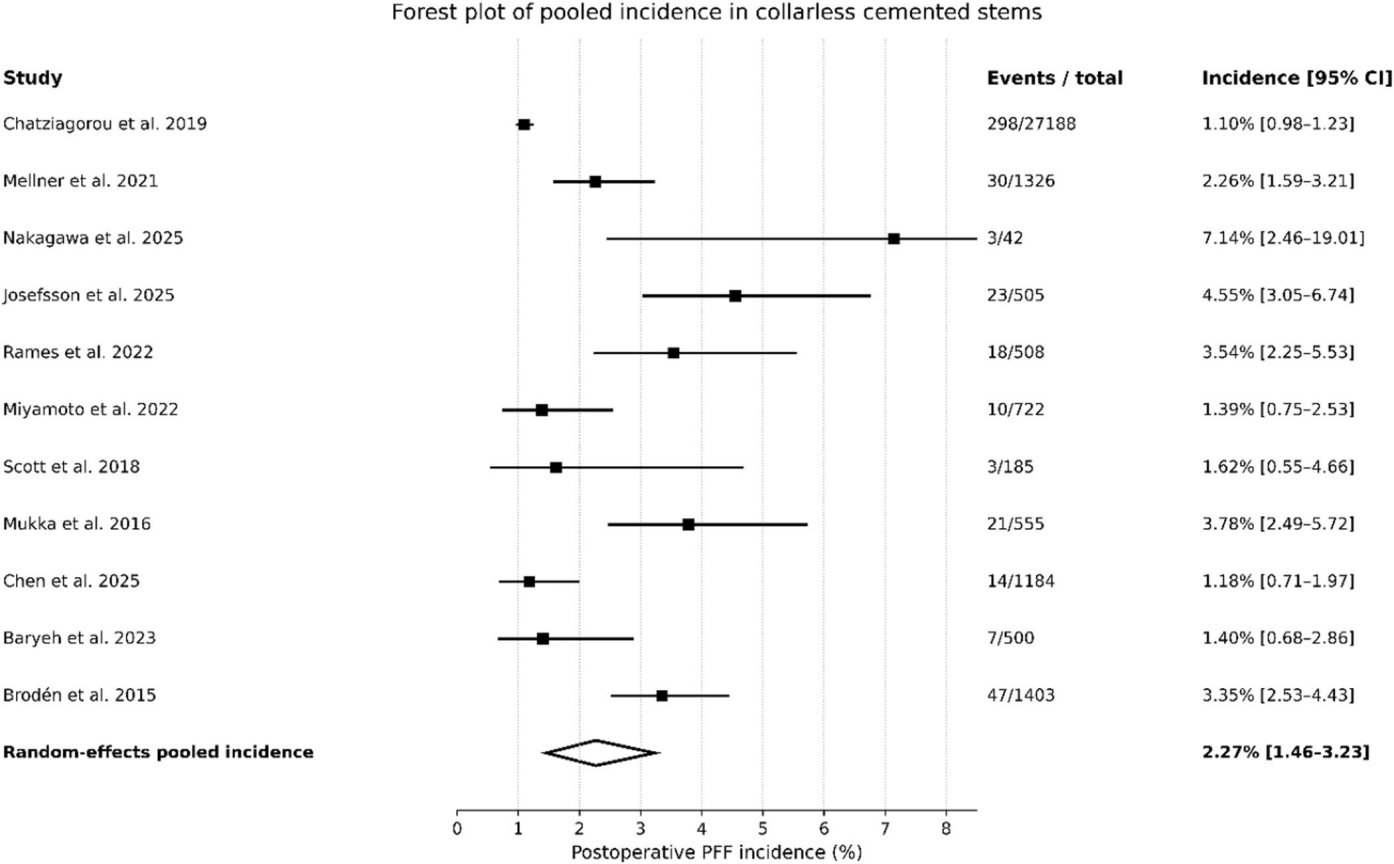
Forest plot of the pooled incidence of postoperative PFF in the collarless cemented stem group. The combined incidence was 2.27%, represented by the diamond. Each line corresponds to an individual study and its CI. CI, confidence interval; PFF, periprosthetic femoral fracture.

### Subgroup analysis by surgical indication

In elective arthroplasty cohorts, the pooled OR for PFF with collared versus collarless stems was 0.30 (95% CI, 0.15–0.63; *p* = 0.0014; *I* ^2^ = 76%). In femoral neck fracture cohorts, the pooled OR was 0.28 (95% CI, 0.20–0.38; *p* < 0.00001; *I* ^2^ = 0%). The test for subgroup differences was not significant (*p* = 0.82) (Figure 5). The pooled incidence of PFF in elective arthroplasty cohorts was 0.45% for collared stems and 1.93% for collarless stems. In femoral neck fracture cohorts, the corresponding incidences were 0.89% and 3.25%, respectively.

**Figure 5:**
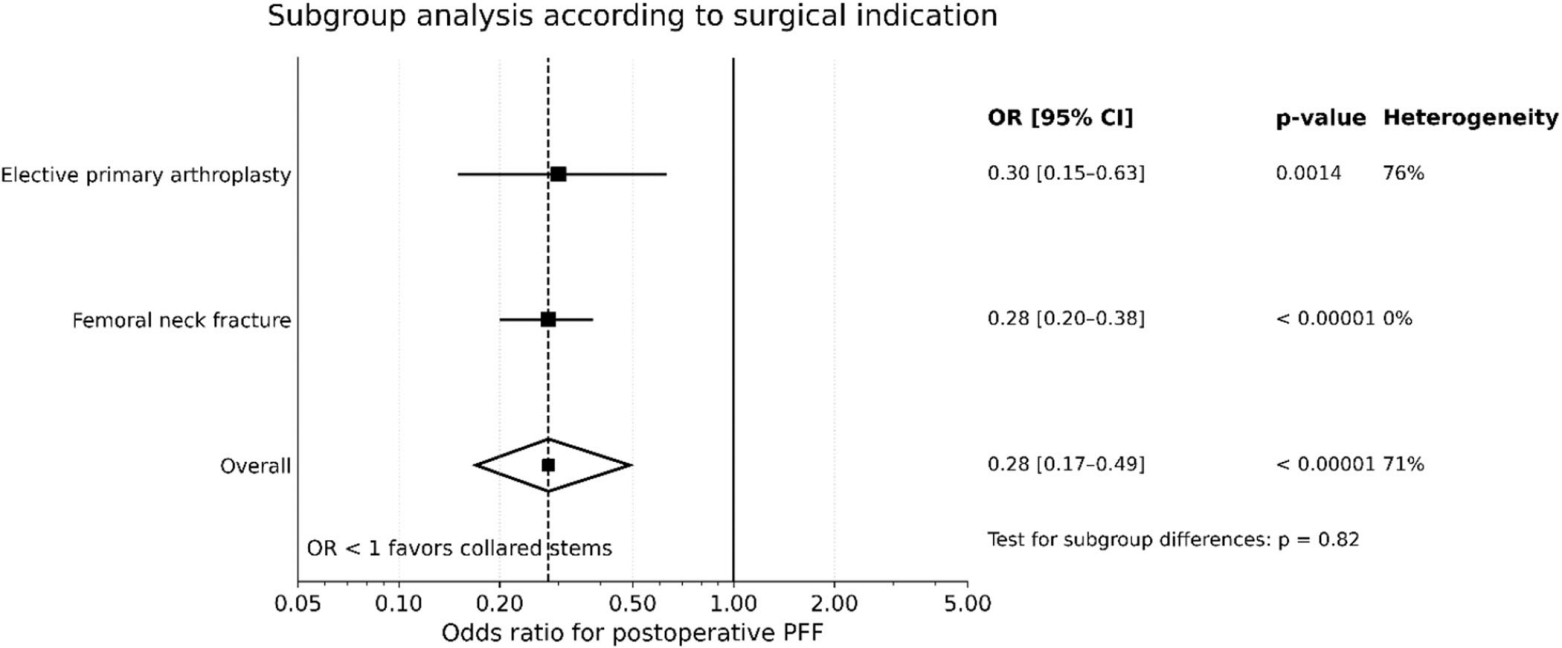
Forest plot of the subgroup analysis according to surgical indication, comparing collared versus collarless cemented stems separately in elective primary arthroplasty and femoral neck fracture cohorts. OR < 1 favours collared stems. The test for subgroup differences did not show a significant interaction between surgical indication and collar status. CI, confidence interval; OR, odds ratio.

### Subgroup analysis by stem surface finish

Only one comparative study directly compared polished collared with polished collarless cemented stems. The OR for postoperative PFF was 0.17 (95% CI, 0.01–2.86; *p* = 0.22) (Figure 6).

**Figure 6:**
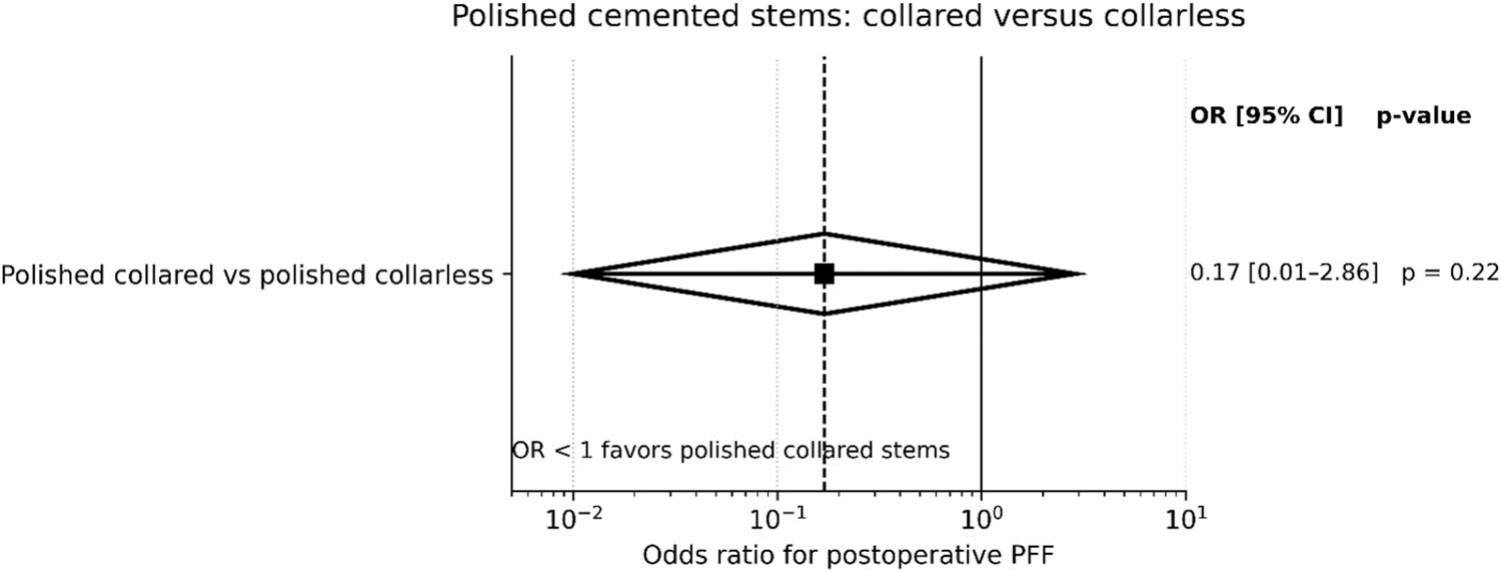
Forest plot of the subgroup analysis restricted to polished cemented stems, comparing polished collared stems with polished collarless stems. This analysis was performed to explore the association of the collar within a more homogeneous surface‐finish subgroup. OR < 1 favours polished collared stems. CI, confidence interval; OR, odds ratio.

Across all available polished‐stem cohorts, including comparative and single‐arm studies, the pooled random‐effects incidence of PFF was 0.65% for polished collared stems and 2.34% for polished collarless stems.

### Vancouver fracture classification

Among fractures with an available Vancouver classification, the distribution of fracture types differed between collared and collarless stem groups (*p* < 0.0001; Figure 7). In the collared group, 16 of 218 fractures (7.3%) were Type A, 60 (27.5%) were Type B, and 142 (65.1%) were Type C. In the collarless group, 11 of 457 fractures (2.4%) were Type A, 338 (74.0%) were Type B, and 108 (23.6%) were Type C.

**Figure 7:**
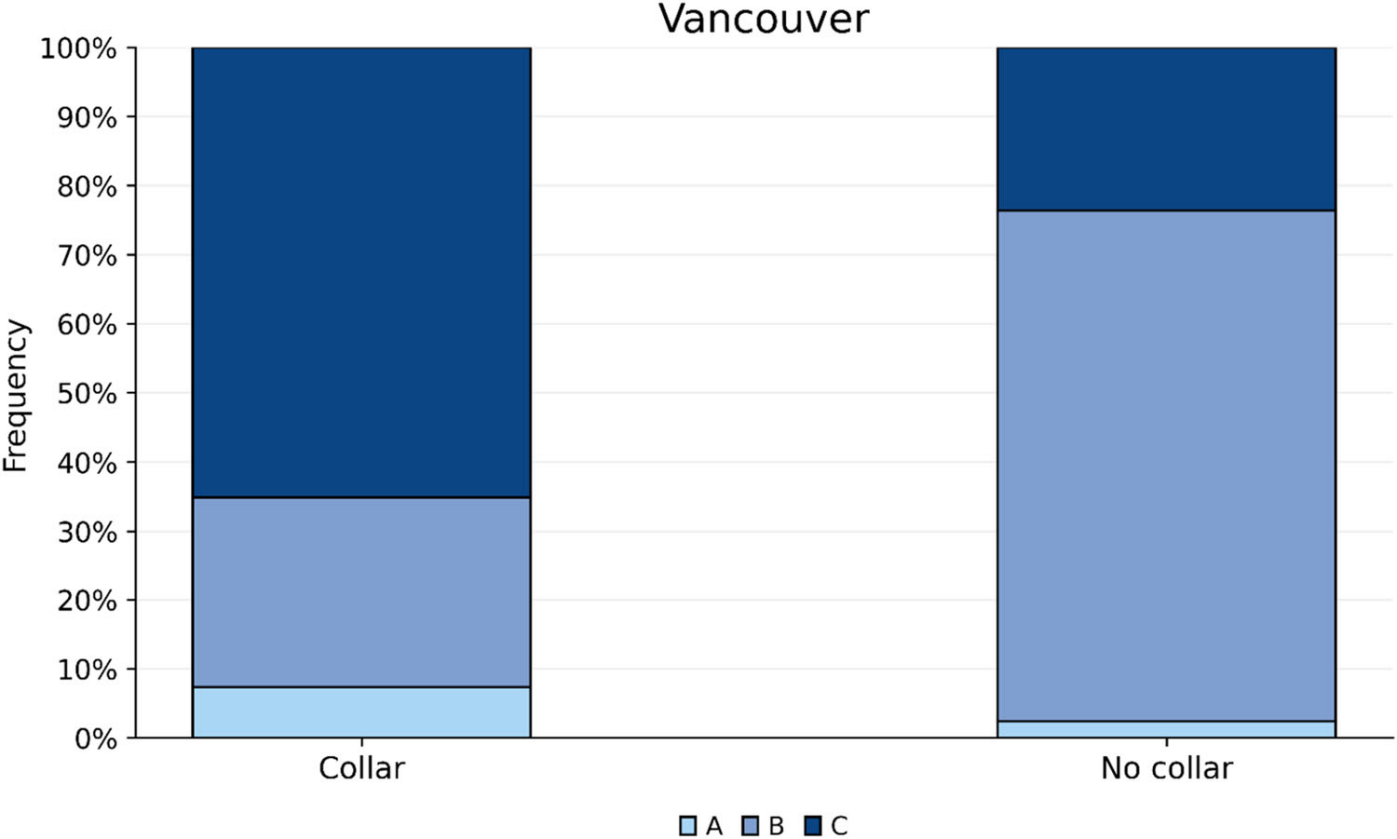
Distribution of Vancouver fracture types between collared and collarless cemented stems. Vancouver C fractures predominated in the collared group, whereas Vancouver B fractures predominated in the collarless group.

### Assessment of publication bias

The funnel plot included nine comparative studies. No formal test of asymmetry was performed. Visual assessment did not reveal obvious asymmetry, but its ability to detect small‐study effects is limited with so few studies; publication bias cannot be excluded (Figure 8).

**Figure 8:**
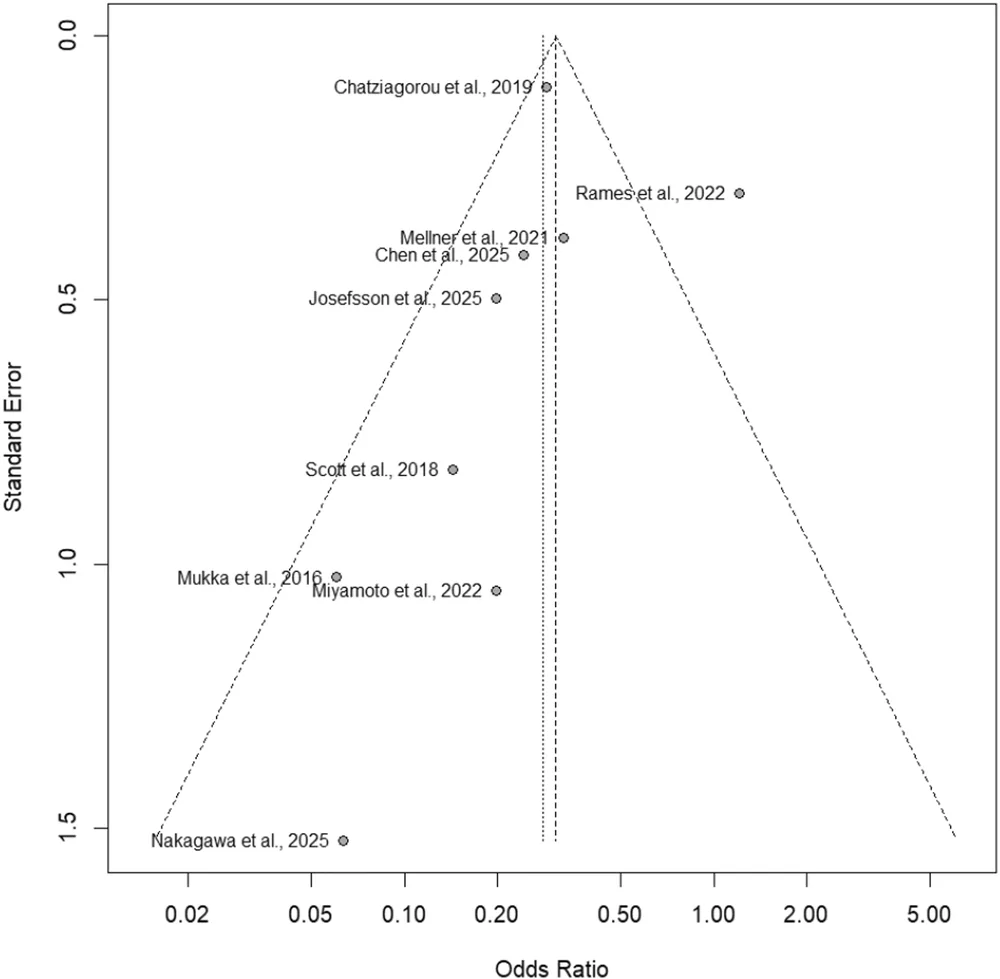
Funnel plot assessing publication bias across the included comparative studies evaluating collared versus collarless cemented stems. No relevant visual asymmetry was observed.

## DISCUSSION

### Primary result

This systematic review and meta‐analysis suggests an association between the presence of a collar and a lower incidence of PFF in cemented stems. Across the comparative studies, the pooled random‐effects OR of PFF with collared versus collarless stems was 0.28 (95% CI 0.17–0.49; *p* < 0.00001) with substantial heterogeneity (*I* ^2^ = 71%). In absolute terms, pooled incidence estimates reinforce the clinical relevance of this difference. The incidence of PFF with collared cemented stems was 0.51% (95% CI 0.22–0.91; *I* ^2^ = 87.8%), whereas collarless cemented stems showed an incidence of 2.27% (95% CI 1.46–3.23; *I* ^2^ = 90.0%).

Predefined subgroup analyses were performed to better account for these potential confounders. When studies were stratified according to surgical indication, the association favouring collared stems was observed both in elective arthroplasty cohorts and in femoral neck fracture cohorts. In elective arthroplasty, the pooled OR was 0.30 (95% CI 0.15–0.63), whereas in femoral neck fracture cohorts the pooled OR was 0.28 (95% CI 0.20–0.38). The absence of a significant subgroup interaction suggests that the lower PFF rate observed with collared stems was not solely driven by the inclusion of frail femoral neck fracture populations. Nevertheless, residual confounding related to age, bone fragility, type of arthroplasty, and indication remains likely and precludes causal inference.

This analysis appears similar to what has been observed with cementless stems, for which the benefit of the collar has been clearly demonstrated [19, 30]. Moreover, Australian Registry data [35] showed higher PFF revision with collarless stems both <6 months (HR 2.90, 95% CI 1.89–4.45) and ≥6 months (HR 10.04, 95% CI 1.38–73.21), indicating that the benefit of a collar may persist beyond early bone ingrowth in cementless fixation. Because collars may remain beneficial even after cementless stem osseointegration, they could also provide calcar support in cemented stems, which are immediately fixed within the femur.

However, this remains a biomechanical hypothesis and should not be interpreted as proof of a causal effect. Nevertheless, available biomechanical studies provide some support for this rationale. Takegami et al. [41] found that the collared Charnley–Kerboull (CK) stem required a higher torque to failure than a collarless polished tapered stem (median 200.5 vs. 164.5 Nm; *p* = 0.046) with fewer Vancouver B‐type fractures. Similarly, Guarin Perez et al. [12] showed that CB stems exhibited greater torque to failure than TS stems (205.3 ± 26.9 vs. 159.5 ± 17.4 Nm; *p* = 0.012) and more distal fracture locations. Taken together, these data support the rationale that a collar on a cemented stem mitigates proximal stress concentration and increases resistance to torsional overload, features that are biomechanically consistent with a lower risk of Vancouver B PFF. This observation was confirmed in the present study with collarless stems, which showed a Vancouver B fracture rate of 74% versus 27.5% for collared stems.

This distinction is relevant as Vancouver type C fractures leave the implant well‐fixed and can be treated with open reduction and internal fixation (ORIF), resulting in lower complication rates than Vancouver B [9, 38].

It is important to note that the present study was not designed to compare TS and CB stems or to isolate the independent effect of surface finish. Although CB stems have been associated with a lower PFF rate than TS stems in a recent meta‐analysis by Mabrouk et al. [23], differences in surface finish, stem geometry and design philosophy make it difficult to determine the specific contribution of the collar in isolation.

This issue is particularly important because, in most available studies, non‐polished stems were collared anatomical or CB designs, whereas collarless stems were usually polished TS designs. Therefore, in non‐polished cemented stems, the effect of the collar cannot be separated from stem morphology, surface finish and fixation philosophy. For this reason, the non‐polished subgroup was considered unsuitable for isolating the specific association of collar status with PFF risk.

To partly address this limitation, a specific subgroup restricted to polished cemented stems was performed, comparing polished collared stems, including CK/French paradox designs, with polished collarless TS stems. This approach reduced confounding related to surface finish, although differences in stem geometry and cementing philosophy remained.

The only direct comparative polished‐stem analysis showed a lower but non‐significant odds of PFF with polished collared stems (OR, 0.17; 95% CI, 0.01–2.86; *p* = 0.22). When all available polished‐stem cohorts were considered, including both comparative and single‐arm cohorts, the pooled random‐effects incidence of PFF was lower for polished collared stems than for polished collarless stems (0.65% vs. 2.34%).

These findings suggest that, even within polished cemented stems, the presence of a collar may be associated with a lower PFF rate. However, this result must be interpreted cautiously because the number of polished collared comparative cohorts was limited, and because polished collared stems are mainly represented by French paradox/CK designs, which differ from collarless polished TS stems in geometry and cementing philosophy.

Beyond the traditional dichotomy TS versus CB, some studies suggest that surface finish may also influence outcomes within similar stem designs. For example, González Della Valle et al. [11] reported lower femoral loosening rates with a polished version of the VerSys stem compared to its matte counterpart, and Hamadouche et al. [13] demonstrated improved long‐term survival of polished versus matte CK stems. In addition, very low rates of postoperative PFF have been reported with highly polished collared stems in specific cohorts, including both standard‐length designs [27], shortened stems [16] and Dorr B/C femurs treated with the French paradox technique [18]. However, it remains difficult to determine whether these low fracture rates are primarily related to the collar itself, to the French paradox cementing technique, or to the interaction between stem geometry, surface finish and cementing philosophy. These findings highlight that stem performance likely depends on multiple interacting factors rather than a single design characteristic [4].

This complexity is further illustrated by the recent study by Chen et al. [7], in which TS stems had a higher revision risk for PFF than CB stems (2.7% vs. 1.2%; adjusted HR, 3.51 [95% CI, 1.46–8.40]), whereas no significant difference was observed in overall aseptic revision risk (HR, 1.38 [95% CI, 0.87–2.20]). Together, these data suggest that fracture outcomes alone may not fully reflect the overall performance of a cemented stem design. Therefore, the respective roles of surface finish and collar are yet to be established and would require dedicated comparative studies.

This study has several limitations. First, all included studies were observational, with an inherent risk of selection bias and residual confounding. The choice of a collared or collarless cemented stem is not random and may be influenced by patient factors known to affect periprosthetic fracture risk, including age, bone quality and proximal femoral morphology such as Dorr type [15]. Second, substantial heterogeneity was observed across studies (*I* ^2^ = 71%), potentially arising from differences in study design, patient populations, surgical indications, stem geometry and surface finish, fixation philosophy and follow‐up duration. This heterogeneity limits confidence in the magnitude and generalizability of the pooled estimate, which should not be assumed to apply to any individual stem design or clinical setting.

Third, the effect of the collar cannot be isolated from other implant‐related and technical factors that may independently influence fracture risk. In particular, stem geometry and design philosophy have been associated with different postoperative periprosthetic fracture risks, while surgical approach and cementation‐related factors such as mantle quality, alignment and technique may also affect femoral loading and fracture behaviour [7]. Although subgroup analyses according to surgical indication and surface finish were performed to address this limitation, they cannot fully eliminate residual confounding, particularly because collar status remains strongly associated with stem design philosophy. Therefore, the findings should be interpreted as demonstrating an association rather than a causal relationship [9].

## CONCLUSION

In this systematic review and meta‐analysis, collared cemented femoral stems were associated with a lower rate of PFF than collarless cemented stems in hip arthroplasty. This association was observed both in elective arthroplasty and femoral neck fracture subgroups, and exploratory analyses restricted to polished stems also suggested lower PFF rates for polished collared stems compared with polished collarless stems. However, substantial heterogeneity and residual confounding limit causal interpretation. The independent effect of the collar remains uncertain and requires further adjusted comparative studies.

## AUTHOR CONTRIBUTIONS

Pierre Laboudie and Mattia Blancatto independently screened studies and assessed study quality; both authors wrote the paper together.

## FUNDING INFORMATION

The authors have no funding to report.

## CONFLICT OF INTEREST STATEMENT

Pierre Laboudie is a consultant for Stryker, Medacta and Amplitude. The remaining author declares no conflict of interest.

## ETHICS STATEMENT

The authors have nothing to report.

## Data Availability

Data extracted from published studies are reported in the article and its tables; additional analysis data are available from the corresponding author upon reasonable request.

## APPENDIX 1

Methodological Quality (Newcastle−Ottawa Scale)

All nine comparative studies achieved high quality scores in Selection, Comparability and Outcome domains, corresponding to an overall ‘Good’ Agency for Healthcare Research and Quality (AHRQ) rating.

**Table:**

Study	Selection	Comparability	Outcome	AHRQ
Chatziagorou et al. 2019	3	2	2	Good
Mellner et al. 2021	3	1	2	Good
Nakagawa et al. 2025	3	1	2	Good
Josefsson et al. 2025	3	2	2	Good
Rames et al. 2022	3	2	2	Good
Miyamoto et al. 2022	3	1	2	Good
Scott et al. 2018	3	1	2	Good
Mukka et al. 2016	3	2	2	Good
Chen et al. 2025	3	2	2	Good

*Note*: Selection domain (max 3 points) assesses cohort definition/representativeness and exposure ascertainment; Comparability (max 2) assesses control of confounders (e.g., age, sex, diagnosis); Outcome (max 2) assesses outcome measurement, follow−up adequacy and losses. According to AHRQ criteria, a total NOS score ≥6 is ‘Good’ quality.
